# Exploring the Relationship between Anti-VEGF Therapy and Glaucoma: Implications for Management Strategies

**DOI:** 10.3390/jcm12144674

**Published:** 2023-07-14

**Authors:** Qëndresë Daka, Nina Špegel, Makedonka Atanasovska Velkovska, Tjaša Steblovnik, Miriam Kolko, Burim Neziri, Barbara Cvenkel

**Affiliations:** 1 Department of Pathophysiology, Medical Faculty, University of Prishtina, 10000 Prishtina, Kosovo; qendrese.daka@uni-pr.edu (Q.D.); burim.neziri@uni-pr.edu (B.N.); 2 Eye Clinic, University Clinical Centre of Kosova, 10000 Prishtina, Kosovo; 3 Department of Ophthalmology, University Medical Centre Ljubljana, 1000 Ljubljana, Slovenia; spegel.nina@gmail.com (N.Š.); makedonka.atanasovskavelkovska@kclj.si (M.A.V.); tjasa.steblovnik@gmail.com (T.S.); 4 Department of Drug Design and Pharmacology, University of Copenhagen, 2100 Copenhagen, Denmark; miriamk@sund.ku.dk; 5 Department of Ophthalmology, Copenhagen University Hospital, Rigshospitalet, 2600 Glostrup, Denmark; 6 Faculty of Medicine, University of Ljubljana, 1000 Ljubljana, Slovenia

**Keywords:** anti-VEGF, elevated intraocular pressure, glaucoma

## Abstract

A short-term increase in intraocular pressure (IOP) is a common side effect after intravitreal anti-VEGF therapy, but a sustained increase in IOP with the development of secondary glaucoma has also been reported in some studies after repeated intravitreal anti-VEGF injections. The aim of this review is to present and discuss the possible pathophysiological mechanisms and factors contributing to a sustained rise in IOP, as well as treatment strategies for patients at risk. Close monitoring and adjustable IOP-lowering treatment are recommended for high-risk patients, including those with glaucoma, angle-closure anomalies, ocular hypertension or family history of glaucoma; patients receiving a high number of injections or at shorter intervals; and patients with capsulotomy. Strategies are needed to identify patients at risk in a timely manner and to prevent sustained elevation of IOP.

## 1. Introduction

Overproduction of vascular endothelial growth factor (VEGF) promotes angiogenesis and induces vascular permeability, contributing to the pathogenesis of several ocular diseases in various ischemic retinal disorders and choroidal neovascularisation (CNV) in age-related macular degeneration (AMD) [1,2,3].

CNV is responsible for severe vision loss in these diseases. Inhibition of VEGF has been proven effective in preventing vision loss and, in some cases, improving vision [2]. However, despite the significant benefits, the use of anti-VEGF drugs is not without potential adverse effects. Over the years, several anti-VEGF drugs have been approved in the form of intravitreal injections, including pegaptanib (Macugen, Eyetech/Pfizer, Inc., Manhattan, NY, USA), a ribonucleic acid aptamer; bevacizumab (Avastin, Genentech, Inc., San Francisco, CA, USA), a recombinant humanised monoclonal antibody; ranibizumab (Lucentis, Genentech, Inc., San Francisco, CA, USA), a humanised monoclonal antibody fragment; aflibercept (Eylea, Regeneron Pharmaceuticals, Inc., Tarrytown, NY, USA), a soluble decoy receptor fusion protein; and the recently launched brolucizumab (Beovu, Novartis, Basel, Switzerland), a humanised monoclonal single-chain variable fragment, and faricimab-svoa (Vabysmo, Genentech, Inc., San Francisco, CA, USA), a bispecific monoclonal antibody that targets both VEGF and angiopoietin 2 (Ang-2) [3,4,5]. 

While RCTs have reported differences in efficacy between these drugs, real-world evidence often fails to observe such distinctions, emphasising the need to consider the limitations and confounders associated with both study types [6,7]. Furthermore, some studies demonstrated nonresponse to this therapy or a loss of efficacy over time [8,9].

The safety profile of anti-VEGF agents is generally favourable, with rare sight-threatening side effects [4,10]. Nevertheless, ocular and systemic adverse effects, such as cardiovascular and renal complications, have been reported, highlighting the importance of cautious use [11,12].

A commonly reported side effect is a transient increase in intraocular pressure (IOP), which usually normalises within 60 min without any intervention [2,13]. In recent years, several studies have reported a sustained elevation of IOP (SE-IOP) [14,15,16,17,18,19], and a few studies have reported the secondary development or progression of pre-existing glaucomatous optic neuropathy (GON) [20,21].

## 2. Methods

We searched the literature to examine the relationship between anti-VEGF therapy and glaucoma in terms of implications for treatment strategies. 

A review was performed to summarise the relevant English-language literature on the development or progression of secondary GON development in patients treated with intravitreal anti-VEGF injections for AMD. The PubMed database was searched using the following terms: “glaucoma development*” OR “glaucoma progression” AND “anti-VEGF intravitreal injections” AND “AMD” OR “age-related macular degeneration”. Articles published up to December 2022 were included in the review without any restrictions based on sex, race or geographic area. Given the different aims and designs of the included studies, a narrative review approach was adopted. 

## 3. Literature Review

AMD and glaucoma are the most common causes of irreversible blindness world-wide [22,23,24]. As the prevalence of AMD increases with age, certain populations are expected to have a higher prevalence of the disease, which is estimated to affect 288 million people worldwide by 2040 [4,25]. With the increasing use of anti-VEGF injections to treat AMD, there is a possibility that the prevalence of developing secondary glaucoma and the rate of progression of pre-existing glaucoma may increase [26].

Currently, this prevalence is unknown because the data come from small, short-term and mostly retrospective studies. We found several studies with very different reports on the association of anti-VEGF treatment and other factors with IOP elevation and the development or progression of GON [3,13,26,27,28,29,30,31]. A meta-analysis of several studies on this topic concluded that the prevalence of SE-IOP was 4.7%, even after accounting for the effects of drug type, disease conditions, follow-up duration, and the exclusion of patients with pre-existing glaucoma and those using corticosteroids [32]. In contrast, the most recent network meta-analysis of eligible RCTs comparing anti-VEGF agents for different retinal diseases found no clear evidence for SE-IOP [26]. However, the authors note that the analysis was limited by imprecision, and no definitive conclusions can be drawn [26].

In addition, data from a large medical database demonstrate an increased risk of initiating IOP-lowering treatment after anti-VEGF injection, although glaucoma patients, glaucoma suspects, individuals with OHT and patients who had received an intraocular steroid injection were excluded [21]. Although data on a direct association with the development of secondary glaucoma and the rate of progression are insufficient [27], a recent retrospective study showed a significant risk [30]. 

The pathogenesis could be multifactorial, as many variables play a role and several theories are thought to explain the underlying mechanisms [10,17,26,27,32].

### 3.1. The Theory of Nitric Oxide 

Nitric oxide (NO) is a crucial signalling molecule involved in various physiological and pathological processes within ocular structures, including vasodilation, neurotransmission and immune response [33]. The detrimental role of NO in GON has been confirmed in many studies that observed changes in the three isoforms of NO synthase (NOS) within ocular structures [33,34,35]. In eyes with normal IOP, NOS-1 was present in astrocytes, pericytes and nerve terminals in the walls of the central artery, and NOS-3 was present in the vascular endothelial cells of both large and small vessels acting as physiological vasodilators in the tissue and neuroprotecting the ONH [34,35,36]. In eyes with elevated IOP, no significant changes in NOS-1 or NOS-3 levels were observed, but NOS-2 appeared in astrocytes in the early stages. NOS-2 produces excessive levels of NO, which is thought to contribute to the neurodestruction of RGC axons by promoting the formation of peroxynitrite and subsequent damage to axons at the lamina cribrosa in the ONH. Furthermore, these studies have shown that inhibition of NOS-2 activity provides protection against the loss of RGCs and preserves their function [34,35,36].

The disruption of NO signalling pathways, particularly through endothelial NOS, by anti-VEGF agents may lead to a decrease in NO levels below physiological baseline, which is thought to be a key mechanism in the development of glaucoma and other processes [20,33,37]. Changes in NO levels may be involved in the pathogenesis of glaucoma through various mechanisms, leading to an increase in IOP, retinal vascular dysfunction and RNFL thinning [23,37]. IOP elevation may be caused by increased resistance in the outflow pathways, increased aqueous humour production in the ciliary body or increased episcleral venous pressure [33,37,38]. Retinal vascular dysfunction could result from impaired vascular autoregulation [33], while RNFL could be affected by the disruption of neuroprotective activities either directly or indirectly through changes in blood flow [38]. While the available data suggest that impaired NO signalling can contribute to glaucoma development through vascular and mechanical mechanisms, increased IOP appears to play a more significant role [33,37]. Several studies have confirmed significant RNFL thinning [38,39,40,41,42,43] and have attributed this to the underlying AMD pathology itself [38,39,40,41] rather than to the anti-VEGF injections [43]. Longitudinal studies over an 8-year period showed no difference in RNFL thickness between injected and control eyes [44], and similar findings were reported in a study involving glaucoma patients receiving anti-VEGF treatment [45]. Furthermore, the relationship between RNFL thinning and progressive visual field loss remains unknown [27]. 

### 3.2. Mechanical Effect of Elevated Intraocular Pressure

Both short- and long-term increases in IOP appear to be causative in the development and progression of glaucoma after anti-VEGF treatment [27,33,39]. The mechanisms responsible for IOP elevation have been discussed in a few studies, and the authors agree that both forms are caused by different mechanisms depending on the underlying pathophysiology and/or ocular conditions [27,33,37,38,39].

Anti-VEGF treatment itself may affect the endogenous expression of VEGF—a paracrine regulator of the conventional outflow pathway [46]. Many studies in humans or animal models have shown a trend towards increased levels of VEGF-A in the aqueous humour of patients with POAG, suggesting a possible neuroprotective role of VEGF in patients with POAG [47].

Brief IOP spikes following anti-VEGF treatment have been attributed to the volume of fluid injected into the eyeball, which can affect mechanical outflow pathways and transiently block axonal transport and ocular perfusion relative to IOP levels, potentially leading to RNFL damage and glaucomatous optic nerve damage [4,13,33,47].

In addition to alterations in the signalling pathways of vasodilatory modulators such as NO, mechanisms thought to be responsible for the development of SE-IOP include pharmacological blockade, damage from trauma and/or IOP spikes, drug-induced inflammation, protein aggregates/silicone oil debris and genomic profile [14,15,16,17,18,19,27,33,48,49,50,51,52,53]. Contributing factors, albeit with conflicting results, are considered to include the type of anti-VEGF agent, treatment interval, number of injections, methods of handling the agent, previous steroid use, glaucoma, angle anomalies, OHT and lens status [14,15,16,17,18,19,27,37,49,50,51,52,53]. SE-IOP appears to be a dominant risk factor for retinal ganglion cell death, RNFL thinning and glaucoma progression [46,47,48,49,50]. 

Anti-VEGFs can temporarily lower the IOP according to their half-life, either by decreasing aqueous humour production in the ciliary body or by mechanically dilating the outflow pathways by matrix metalloproteinase (MMP) activity. However, the IOP may increase due to rebound swelling of the cells in the outflow pathways or a renewed production of aqueous humour in the ciliary body [51].

### 3.3. The Role of Anti-VEGF Agents

Although results have been contradictory, studies have pointed to differences between anti-VEGF agents that may be due to either molecular properties, pharmaceutical preparation, storage or method of administration. However, it is difficult to determine which agent carries a higher risk, as patients usually receive several different agents during their treatment. The studies have mainly looked at bevacizumab, followed by ranibizumab, and less at aflibercept. Anti-VEGF agents can cause SE-IOP and, consequently, GON by different mechanisms based on their properties. Bevacizumab is considered to have a higher risk of causing SE-IOP by any mechanism. 

#### 3.3.1. Pharmacological Blockade

All molecules can accumulate in the outflow pathways and cause direct mechanical obstruction or an indirect physiological change in outflow [18,51,52]. Bevacizumab (149 kDa) is considered to have a higher risk due to its molecular weight and longer half-life, followed by aflibercept (115 kDa), ranibizumab (48 kDa), brolucizumab (26 kDa) and pegaptanib (20 kDa) [3,17,52]. However, some studies found no difference between the agents [18,53] or a higher prevalence for lower-molecular-weight ranibizumab compared to aflibercept [31,54]. 

#### 3.3.2. Contamination

Outflow obstruction can result from protein aggregates and/or silicone droplets from syringes, freezing/thawing, exposure to light, mechanical shock, improper storage or administration of the anti-VEGF agent [50,55]. In addition, a number of other materials may enter the protein solution, including ions, plasticisers and other organic molecules [55]. An increase in these proportions may lead to SE-IOP due to mechanical effects, toxicity or immunity [55]. This theory is supported by the fact that in some cases, including ours, the increase in IOP could only be controlled after filtration surgery, as was the case with silicone-oil-induced glaucoma, and that SE-IOP was not observed when silicone-free syringes were used [28,50]. However, in a prospective study, silicone oil droplets were not observed in the anterior chamber [51], and no association was found between the number of injections with protein aggregates in their packaging and SE-IOP [53]. Bevacizumab is thought to have an increased risk because it is drawn from a larger vial, usually in multiple syringes not designed for protein products, and frozen for a variable time compared to single-dose vials drawn into the syringe immediately before injection [50,55]. However, the source appears to play a greater role than the drug itself, as differences in SE-IOP were found in eyes treated with repackaged bevacizumab from different suppliers [3,19,40,55].

A direct toxic effect of anti-VEGF drugs on the outflow pathways seems unlikely [3,50]. Only bevacizumab was found to be toxic to TM cells, and only when the concentration was four times higher than the clinical dose [3,40]. However, the toxic effect could be caused by impurities [55].

#### 3.3.3. Inflammation

Inflammation can obstruct aqueous humour outflow by causing scarring and the proliferation of fibroblasts [3,52]. Theoretically, it can have different causes: subclinical inflammation after injection related to an immunological response to monomeric antibodies, especially to contaminants; chronic inflammation related to repeated injections and transient angle closure; or trabeculitis caused by the pharmacological molecule itself [15,33,49,50,51,52,55,56,57]. The risk of severe intraocular inflammation is increased 12-fold with bevacizumab compared with ranibizumab, probably due to the proinflammatory Fc component and the longer half-life of large antibodies [3,17,58]. Although many studies failed to demonstrate anterior chamber cells, flare, synechiae or trabeculitis [17,19,51], and some found that treatment with topical corticosteroids did not control inflammation or lower the IOP [51], the cause could still be low-grade inflammation that cannot be detected via slit-lamp examination [59,60].

### 3.4. The Role of the Number of Injections and Intervals between Them

The total number of injections and interval regimen may be considered as independent causal factors for the pharmacological agent, as a significant correlation of SE-IOP with the number of injections, especially if more than 20, and intervals of less than 8 weeks has been demonstrated [10,15,16,19,30,46,51,59,61]. In studies, eyes treated with more than 20 anti-VEGF injections were found to have up to a 12% reduction in aqueous humour outflow [46], while patients in whom the interval was increased were found to have a lower SE-IOP score [28] and reduced need for IOP treatment [51]. The number and interval of injections have also been associated with the risk of initiating IOP-lowering treatment for secondary glaucoma [21] and glaucoma surgery [62]. However, no significant associations were found in some studies [17,19,52,53].

### 3.5. The Role of Glaucoma, OHT and Angle Anomalies

An already compromised outflow system is thought to be a contributing factor for SE-IOP due to the disruption of endogenous VEGF signalling involved in outflow regulation. While a family history of glaucoma [16], compromised angles including narrow angles [20], angle synechiae, heavy trabecular pigmentation [61] and OHT [46] are associated with increased risk of SE-IOP, glaucoma itself appears to be an independent risk factor [17,27,32]. Studies found a significant decrease in tonographic outflow in OHT patients [17,46] and the development of up to 50% SE-IOP after less than 10 injections in glaucoma patients [17,52]. Studies that did not find any association pointed out their inclusion of a low number of glaucoma patients or no inclusion at all [15,16,18,19,51].

### 3.6. The Role of Lens Status

The relationship between lens status and the development of SE-IOP after anti-VEGF injection appears to be complex [3]. Some studies showed no association between lens status and SE-IOP [15,46], others suggested that phakic eyes or pseudophakic eyes may be risk factors after capsulotomy [16,30,52,53,61], and a few studies showed a prophylactic role of phakic lens status [18,30]. 

Although cataract extraction is known to lower IOP, pharmacokinetic studies of anti-VEGF agents have shown increased diffusion into the anterior chamber and increased clearance after lensectomy/vitrectomy [30,63,64,65]. In addition, disruption of the lens capsule, anterior hyaloid or zonules allows contaminants to enter the anterior chamber, exposing pseudophakic patients and patients undergoing laser capsulotomy to an increased risk of developing SE-IOP [3,52]. On the other hand, the increased risk of SE-IOP and glaucoma development in phakic patients is explained by the mechanical effect of pressure shifting the lens–iris diaphragm anteriorly and compressing the anterior chamber volume, leading to outflow pathway strain [30]. In pseudophakic patients, this strain can be reduced by both faster volume equilibration and faster resolution of IOP after injection due to the more open anterior chamber [30,45].

### 3.7. The Role of Steroid Treatment

Previous intravitreal steroid injection is listed among the risk factors for SE-IOP after anti-VEGF injection, although some studies have shown no association [3]. The association has not been studied, but it has been suggested that a common pathway is responsible [51], as the effect of steroids on extracellular matrix deposition in the TM, aqueous humour dynamics and gene expression has been demonstrated [3,60]. The same effect was also observed in patients treated with systemic and/or topical steroids who experienced a more rapid and severe increase in intraocular pressure requiring aggressive IOP-lowering treatment [3,60].

## 4. Conclusions and Management Strategies

Causal relationships regarding the development and/or progression of glaucoma remain very difficult to study due to glaucoma’s interaction with retinal diseases [20,30]. SE-IOP associated with anti-VEGF treatment remains the main risk factor, and its mechanical effect seems to be more important for the pathogenesis of the disease [30,37]. However, the effect of elevated IOP on retinal ganglion cells and RNFL damage may be exacerbated by the ability of anti-VEGF agents to negatively affect blood flow in the retina and optic disc [33].

The pathogenesis of SE-IOP is not clear, and there have been few studies investigating genetic, molecular and protein alterations in the outflow pathways [3,60]. A reduction in aqueous humour dynamics in eyes that have received a higher number of anti-VEGF injections has been confirmed [46], but further studies are needed to clarify the pathophysiology and quantify the potential association between short- or long-term IOP elevations and the development of secondary glaucoma or progression of pre-existing glaucoma [27,51,52,53]. In addition, data are limited for the newer anti-VEGF agents aflibercept, brolucizumab, and faricimab.

Timely detection of SE-IOP appears to be important to delay the disease, but strategies to identify patients at risk should be explored [27]. While literature data suggest that lowering the IOP prior to treatment is beneficial in preventing IOP spikes, there is no consensus on protocols to prevent SE-IOP, while the impact on the development or progression of glaucoma is unknown [20,26,27]. The available data recommend close monitoring and prescription of medications to lower IOP in high-risk patients, including those with glaucoma, angle anomalies, family history of glaucoma or OHT; patients receiving a high number of injections or at shorter intervals; and patients with capsulotomy [3,19,39,46,57]. To prevent immediate postoperative elevation of IOP in glaucoma patients, the French Glaucoma Society suggests the instillation of 1% apraclonidine or dorzolamide/timolol a few hours before anti-VEGF injection [66], while prophylactic administration of acetazolamide 60–90 min before intravitreal ranibizumab injection showed a statistically significant but modest reduction in IOP after 30 min [67]. Currently, predicting the likelihood of a complication in the other eye is uncertain [53], and monitoring pRNFL thickness is not a standard procedure, although it seems reasonable to consider in eyes at risk of glaucoma [20,27]. Switching patients to a “pro re nata” regimen, using longer-acting agents and avoiding syringes that risk leaving particles [3,20,28,30,35,51,54,57] have been suggested as ways to avoid SE-IOP. However, it is believed that treatment with a lower frequency and higher potency is beneficial if the effect is actually related to the injection event rather than pharmacological blockade [23]. Iridotomy may be an effective preventive measure in hypermetropic eyes [30], and considerations are recommended when performing capsulotomy in patients on anti-VEGF treatment [52]. Some studies recommend modifying the injection technique [3]. Treatment of SE-IOP is required and can be performed with topical or systemic IOP-lowering drugs, laser treatment or, rarely, filtration surgery [51,57]. It is thought that better control can be achieved with NO donors as they directly target the pathophysiological decrease in NO [33]. Therefore, pharmacological neuroprotection by NOS-2 inhibition, such as the use of aminoguanidine or blocking NOS-2 induction and gene expression, may be a promising approach for the treatment of patients with glaucoma. By protecting the axons at the level of the ONH from neurodegeneration caused by chronic, moderately elevated IOP, NOS-2 inhibition has the potential to prevent the loss of retinal ganglion cells [34,35].

In some cases, IOP levels can be stabilised after switching to a “pro re nata” regimen, suggesting that a longer interval may allow for the elimination of the drug from the eye [51,57].

Further research is required to comprehensively understand and quantify the risk of developing or progressing to glaucoma associated with anti-VEGF treatment. Although a few studies have investigated the relationship between anti-VEGF treatment and the development or progression of glaucoma using visual field analysis, pRNFL thickness measurements and optic nerve analysis, the data available to date are inconclusive [21,27,62].

## Data Availability

Not applicable.
